# Is *Duhuo Jisheng Tang *containing *Xixin *safe? A four-week safety study

**DOI:** 10.1186/1749-8546-5-6

**Published:** 2010-02-11

**Authors:** Shu-Ching Hsieh, Jung-Nien Lai, Pau-Chung Chen, Chao-Chung Chen, Huey-Jen Chen, Jung-Der Wang

**Affiliations:** 1 Division of Health Technology Assessment, Center for Drug Evaluation, Taipei 100, Taiwan; 2 Institute of Occupational Medicine and Industrial Hygiene, College of Public Health, National Taiwan University, Taipei 100, Taiwan; 3 Department of Obstetrics and Gynecology, Department of Chinese Medicine, Taipei City Hospital, Yangming Branch, Taipei 100, Taiwan; 4 Institute of Traditional Medicine, School of Medicine, National Yangming University, Taipei 100, Taiwan; 5 Division of Traumatology, Chinese Medicine Branch, Taipei City Hospital, Taipei 100, Taiwan; 6 Division of Chinese Internal Medicine, Chinese Medicine Branch, Taipei City Hospital, Taipei 100, Taiwan; 7 Department of Internal Medicine, National Taiwan University Hospital, Taipei 100, Taiwan; 8 Department of Environmental and Occupational Medicine, National Taiwan University Hospital, Taipei 100, Taiwan

## Abstract

**Background:**

Though the nephrotoxicity and carcinogenicity of aristolochic acid (AA) are known, its safety in clinical usage is not clear. This study aims to evaluate the safety of *Duhuo Jisheng Tang *(DJT) in a four-week study to treat osteoarthritis (OA) of the knee.

**Methods:**

A qualitative and quantitative investigations on DJT were conducted. A list of adverse events (AEs), complete blood counts, and liver and kidney function tests were measured for participants with knee OA at their scheduled hospital visits. Each detected AEs was independently assessed for severity and causality by site investigators (Chinese medical doctors) and study nurses.

**Results:**

A total of 71 eligible subjects were included in the clinical study where 287 AEs were reported. DJT did not contain detectable aristolochic acid (AA) under thin-layer chromatography (TLC) analysis and gas chromatography coupled with mass spectrometry (GC-MS). There were no significant changes in liver or kidney functions.

**Conclusion:**

In four-week use of DJT, no renal tubular damage, no severe incidences of AEs and adverse drug reactions (ADRs) were observed. The present study obtained safety data from active surveillance of DJT.

## Background

While medicinal herbal products are widely used [1,2] with a presumption that natural herbs are safe, there is a lack of safety evidence to support such products. Since the discovery of nephrotoxicity and carcinogenicity of aristolochic acid (AA) [3-5], the International Agency for Research into Cancer (IARC) has considered herbal remedies containing plants of *aristolochia *genus as Group 1 human carcinogens and those containing naturally occurring mixtures of AAs as 2A carcinogens [6]. Medicinal plants that contain AAs are banned in certain countries, including USA, UK, Canada, and Taiwan [7,8].

*Xixin (Radix et Rhizoma Asari*), also known as *Saishin *in Japan or *Sesin *in Korea, is widely used in many parts of Asia despite that it contains AAs [9-11]. For example, since 2004, a total of 393 Chinese herbal products (CHPs) containing *Xixin *have been reimbursed under the National Health Insurance (NHI) in Taiwan [12] where the regulations stipulate that AA must be undetectable in final herbal products [13]. Our preliminary analysis of NHI data found that about 1.57 million in Taiwan have been prescribed with CHPs containing *Xixin*. *Duhuo Jisheng Tang *(DJT), an herbal formula described by the ancient Chinese physician *Sun Simiao *in 652 AD to treat low back/knee pain [14-16], was prescribed to 725,549 patients between 1996 and 2004. DJT was attributed to AA-related nephropathy in a case report [17].

In another aspect, many clinical trials of Chinese herbal medicines (CHMs) have been rated as having poor methodological quality [18], though CHMs is regarded by the World Health Organization (WHO) as in the urgent need to establish evidence-based information [19]. The active monitoring of safety profile demonstrated in our previous study is proved useful to supplement the current pharmacovigilance function [20]. Therefore, the present study aims to determine whether AA is present in *Xixin*-containing CHPs such as DJT and whether DJT use causes acute nephrotoxicity by qualitative and quantitative methods incorporated in active safety surveillance system. The results of efficacy evaluation were reported in a separate paper [21].

## Methods

### DJT preparation

The investigational DJT was supplied by Sun Ten Pharmaceutical Company (Taiwan) in a standardized form of 15 concentrated herbal extracts, namely *Duhuo *(*Radix Angelicae Pubescentis*), *Qinjiao (Radix Gentianae Macrophyllae), Fangfeng (Radix Saposhnikoviae)*, *Xixin*, *Rougui (Cortex Cinnamomi)*, *Sangjisheng (Herba Taxilli)*, *Duzhong (Cortex Eucommiae)*, *Shudihuang (Radix Rehmanniae)*, *Niuxi (Radix Cyathulae)*, *Danggui (Radix Angelicae Sinensis)*, *Baishao (Radix Paeoniae Alba)*, *Chuanxiong (Rhizoma Chuanxiong)*, *Renshen (Radix et Rhizoma Ginseng)*, *Fuling (Poria) *and *Gancao (Radix et Rhizoma Glycyrrhizae)*. The final product is 2.5 g of granules packed in a sachet.

The investigational DJT, three other brands of each of DJT and *Xixin *were produced by pharmaceutical companies with good manufacturing practice (GMP) certification. All samples were sent to the Bureau of Food and Drug Analysis of Taiwan for chemical analysis. Thin-layer chromatography (TLC) and gas chromatography coupled with mass spectrometry (GC-MS) were used to confirm *Xixin *in these samples by identifying their characteristic peaks of Asarinin. High-performance liquid chromatography (HPLC) and liquid chromatography coupled with tandem mass spectrometer (LC/MS/MS) were then carried out to detect AA in these samples [22].

A clinical study with active safety surveillance [20] on detection and causality assessment of adverse events (AEs) was launched at the Yangming and Chinese Medicine Branches of Taipei City Hospital. This study was approved by the Joint Institutional Review Board for Traditional Chinese Medicine, and received regular external monitoring and auditing by a third Research Organization during the study period.

### Inclusion and exclusion criteria for study subjects

Adult patients with osteoarthritis in at least one knee in the previous six months were recruited for the study. Exclusion criteria are as follows: (1) intra-articular injections of non-steroidal anti-inflammatory drugs within one month; (2) secondary arthritis related to syphilitic neuropathy, ochronosis, metabolic bone disease or acute trauma, severe osteoporosis (≥ grade 3) [23] or acute rheumatic arthritis; (3) significant co-morbidities, such as hypertension, severe hepatitis, kidney diseases or malignant carcinoma; (4) spinopathy caused by tumor (benign or malignant); (5) the use of any other investigational drugs within the past 30 days; (6) women with childbearing potential who had not used adequate contraception since their last menstruation or would not continuously use adequate contraception during the study period; and (7) women who were lactating or positive in a urine pregnancy test within 14 days prior to the study. Quota for recruiting participants from Yangming and Chinese Medicine Branches of Taipei City Hospital was 40. All recruited subjects were required to sign an informed consent form and to discontinue any form of treatment and current medications, including western medicine and CHPs, at least two weeks prior to the screening for the study.

### Active surveillance for safety

A surveillance list of 20 pre-hypothesized AEs was compiled with spaces allowed for AEs not in the list. The AE-specific form with the description of the signs and symptoms was used to ask subjects questions for causality information [24], including necessary criteria (temporality), quasi-necessary criteria (consistency, chance elimination and confounders as alternative explanations and coherence with other highly corroborated theories) and other supportive criteria (e.g. strength and specificity of association, dose response relationship and biological plausibility). The information on every reported AE was collected in respective structured forms for causality assessment. Through a prospective design, we collected the quantitative and qualitative data related to each AE and looked for possible explanation during each incidence. Moreover, we adopted a consensual approach to minimize disagreements and reach a more credible conclusion in causality assessment for AE-drug combinations [25,26].

The recruited subjects were required to take two sachets (5 g) of the investigational DJT per day for four weeks, an adequate treatment course estimated by the clinicians for the measurement of DJT efficacy and for the detection of early tubular damage to the kidney. Laboratory tests were conducted at baseline visit and four weeks after taking DJT, including routine urinalysis, complete blood and platelet counts, biochemistry measures and urinary N-acetyl-glucosaminidase (NAG) and retinal binding protein (RBP) [27,28].

Every detected/reported AE was independently assessed by onsite investigators (Chinese medical doctors) and study nurses. The investigators applied unstructured clinical judgment [29] to rate the AEs severity as mild (transient or mild discomfort; no medical intervention required), moderate (some assistance needed; no medical intervention required) or severe (marked limitation in activity; medical intervention required and possible hospitalization); and rate the AE-drug causality as uncertain, probable/likely, possible, unlikely, conditional/unclassified and unassessable/unclassifiable [30]. The study nurses adopted the Common Terminology Criteria for Adverse Events (CTCAE) v3.0 [31] to judge the severity of the AEs and Naranjo scale [32] to assess causality. Any severe AEs, or any AEs judged to be highly correlated to DJT was be submitted to the research panel for final decision.

### Data analysis

The data were analyzed using simple statistics. The outcome variables were the incidence rates of AEs and adverse drug reactions (ADRs), which were calculated by dividing the event counts of the AEs and ADRs (the numerator) by the exposure level, in terms of person-days or person-sachets (the denominator). The former was the total number of all the participants' exposed (intend-to-treat) person-days to the study drug, while the latter was the actual number of sachets which participants had taken during the study period.

## Results

### Laboratory tests

TLC results revealed that seven samples contained *Xixin*. GC-MS results also confirmed Asarinin, the main component of *Xixin*. LC/MS/MS or HPLC detected AAI and AAII only in the three brands of *Xixin *samples when those were condensed 25 times (Table 1).

**Table 1:** Detection of aristolochic acid (AA) in study drug and other brand products in the market

		Contents (μg/g)	
		
Chinese herbal products	Sample no.	AA-I^a^	AA-II^a^
DJT (study drug)	1	(--)	(--)
	25-fold concentration	(--)	(--)
Other brand products bought in the open market			
DJT (brand 1^b^)	2	(--)	(--)
	16.7-fold concentration^c^	(--)	(--)
DJT (brand 2)	3	(--)	(--)
	25-fold concentration	(--)	(--)
DJT (brand 3)	4	(--)	(--)
	10-fold concentration^c^	(--)	(--)
*Xixin *(brand 1^b^)	5	(--)	(--)
	25-fold concentration	6.64	(--)
*Xixin *(brand 2)	6	(--)	(--)
	25-fold concentration	6.77	(--)
*Xixin *(brand 3)	7	(--)	(--)
	25-fold concentration	9.95	(--)

Notes:

a The detection limit of aristolochic acid I (AA-I) and aristolochic acid II (AAII) is 2.0 ng/ml

^b^Brand 1 products (samples nos. 2 and 5) were also produced by Sun Ten Pharmaceutical Company (Taiwan), but they were directly purchased from the market.

^c^Samples nos. 2 and 4 could only be concentrated up to less than 25-fold during the test procedure.

### Subjects

**Figure 1:**
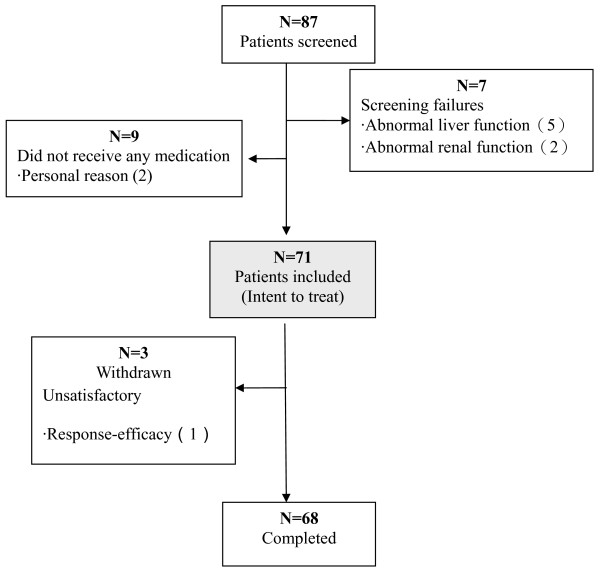
**Recruitment of participants' inclusion, withdrawal, and completion during follow-up**.

A total of 87 subjects who signed the informed consent form were screened. Sixteen subjects did not receive medication due to various reasons (Figure 1). Intent-to-treat monitoring was given to all the rest of 71 subjects.

### Occurrences of adverse events

None of the subjects showed any abnormality of urinalysis, creatinine or blood urea nitrogen, NAG or RBP. A total of 287 AEs were detected/reported during the study period (Additional file 1) and were coded according to the Coding Symbols for Thesaurus of Adverse Reaction Terms (COSTART) [33]. The most often reported AEs were rashes, abdominal fullness, coughs, somnolence, muscle cramps and diarrhea with the incidence rates of 14.5, 12.9, 12.4, 11.9, 10.3, and 10.3 per 1000 person-days respectively; and 7.5, 6.9, 6.6, 6.3, 5.5, 5.5 per 1000 person-sachets respectively. All of these AEs were monitored according to the original surveillance list. No additional AE was found.

### Severity of the adverse events and association with DJT

All recorded AEs were classified as 'mild' to 'moderate' according to the investigators (Chinese medical doctors), and as 'Grade 1' or 'Grade 2' according to the study nurses. These AEs were tolerable and did not have any significant effects on the subjects' daily activities. The probable ADRs separately detected by the investigators (Chinese medical doctors) and study nurses are summarized in Table 2.

**Table 2:** Probable adverse drug reactions detected by the panel of investigators (Chinese medical doctors) and study nurses

Cases adjudged by the panel to be probable ADRs	Judgment by study nurses under the Naranjo scale	Subjective judgment of the investigators	Risk per 10^3 ^person-days^a^	Risk per 10^3 ^person-sachets^a^
Change in skin color	Probable	Probable/Likely	0.5	0.3
Red flush	Probable	Certain	0.5	0.3
Diarrhea	Possible	Probable/Likely	0.5	0.3
Tachycardia	Probable	Unlikely	0.5	0.3

Notes:

^a^The number of cases was used as the numerator for the calculation of the risk. The denominators for the calculation of the risks were 1,936 person-days and 3,633 person-sachets.

## Discussion

In the present study, AA was undetectable in the investigational DJT and three other brands of DJT, whereas the main peaks of the active ingredients (e.g. Asarinin) of *Xixin *were present in the samples. This result supports the regulations set by the Committee on Chinese Medicine and Pharmacy of Taiwan, which stipulates that only the root portion of *Xixin *can be used for herbal products, and therefore the final product should not contain any detectable AA [13]. However, care must be taken to prevent people from using raw herbs of *Xixin *which may contain higher levels of AA.

The possible existence of AA in DJT may pose a potential hazard to patients' health [34-37]. We attempted to indirectly estimate the average levels of AA in DJT from concentrated *Xixin*. As shown in Table 1, AAI was detected only in concentrated (25 times) *Xixin *products. Those subjects who completed the study would have ingested a maximum daily dosage of 0.04-0.08 μg of AAI, which was a total of 1.34-2.01 μg for the study period. None of these subjects showed any significant renal tubular damage.

A four-week study of *Duhua Jisheng Wan *[38], which has the same ingredients of DJT, also did not result in any kidney-related damage. As compared to the reported case of the AA-related nephropathy patient [17] ingesting 400 g of DJT powder for over four months, the ingestion levels of AA in this study were very low. However, the holistic effect stressed by CHMs theory [16] in mixing different herbs as a meaning of enhancing efficacy and minimizing toxicity cannot be completely ruled out. To evaluate the overall safety of the final DJT product as a formulated herbal preparation of 15 herbs, we list AEs for DJT in additional file 1.

The absence of a control group makes the observed results from the present study subject to potential confounders. To minimize confounders, we implemented two independent assessments by site investigators (Chinese medical doctors) and study nurses, respectively. In addition, the sample size of the present study is small.

## Conclusion

In four-week use of DJT, no renal tubular damage, no severe incidence of AEs and ADRs were observed. The present study obtained safety data from active surveillance of DJT.

## Abbreviations

DJT: *Duhuo Jisheng Tang*; AA: aristolochic acid; OA: osteoarthritis; GCP: good clinical practice; AEs: adverse events; ADR: adverse drug reactions; TLC: thin-layer chromatography analysis; GC-MS: gas chromatography coupled with mass spectrometry; CHPs: Chinese herbal products; NHI: National Health Insurance; CHMs: Chinese herbal medicines; IARC: International Agency for Research into Cancer; GMP: good manufacturing practice; HPLC: high-performance liquid chromatography; LC/MS/MS: liquid chromatography coupled with tandem mass spectrometer; NAG: N-acetyl-glucosaminidase; RBP: retinal binding protein; CTCAE: Common Terminology Criteria for Adverse Events; WHO: World Health Organization.

## Competing interests

The authors declare that they have no competing interests.

## Authors' contributions

SCH performed the study design and statistical analysis, monitored the study, and prepared the manuscript. JNL designed the study and assisted in patient recruitment with assistance from HJC and CCC. PCC conducted the statistical analysis and data interpretation. JDW conceived, designed and coordinated the study, and drafted the manuscript. All authors read and approved the final manuscript.

## Supplementary Material

Additional file 1 **Summary of adverse event data**. Table summarizing adverse events for Duhuo Jisheng Tang. Click here for file
