# Supplementary material for: Is Duhuo Jisheng Tang containing Xixin safe? A four-week safety study
Source: Chin Med. 2010 Feb 11;5:6. doi: 10.1186/1749-8546-5-6 (PMC2829565; doi:10.1186/1749-8546-5-6)
Supplement: Additional file 1 — Summary of adverse event data. Table summarizing adverse events for Duhuo Jisheng Tang. [file 1749-8546-5-6-S1.DOC]

Additional file 1

Summary of adverse event data

| **Adverse events ^a^** | **No. of cases** | **No. of prior occurrences** | **Risk per 10^3^ person-days ^b^** | **Risk per 10^3^ person-sachets ^b^** |
| --- | --- | --- | --- | --- |
| Body as a whole |  |  |  |  |
| Asthenia | 2 | 2 | 1.0 | 0.6 |
| Chest pain | 6 | 5 | 3.1 | 1.7 |
| Abdominal fullness | 25 | 12 | 12.9 | 6.9 |
| Abdominal pain | 16 | 9 | 8.3 | 4.4 |
| Fever | 2 | 2 | 1.0 | 0.6 |
| Headache | 9 | 7 | 4.6 | 2.5 |
| Neck rigidity | 1 | 1 | 0.5 | 0.3 |
| Pain | 8 | 7 | 4.1 | 2.2 |
| Nervous system |  |  |  |  |
| Dizziness | 4 | 2 | 2.1 | 1.1 |
| Dry mouth | 8 | 8 | 4.1 | 2.2 |
| Insomnia | 2 | 1 | 1.0 | 0.6 |
| Somnolence | 23 | 13 | 11.9 | 6.3 |
| Tremor | 1 | 0 | 0.5 | 0.3 |
| Cardiovascular system |  |  |  |  |
| Bradycardia | 2 | 1 | 1.0 | 0.6 |
| Flashes | 1 | 1 | 0.5 | 0.3 |
| Hypertension | 1 | 1 | 0.5 | 0.3 |
| Hypotension | 7 | 3 | 3.6 | 1.9 |
| Tachycardia | 1 | 1 | 0.5 | 0.3 |
| Digestive system |  |  |  |  |
| Constipation | 7 | 5 | 3.6 | 1.9 |
| Diarrhea | 20 | 15 | 10.3 | 5.5 |
| GI disease | 2 | 2 | 1.0 | 0.6 |
| Nausea | 6 | 3 | 3.1 | 1.7 |
| Ulcer mouth | 3 | 3 | 1.5 | 0.8 |
| Vomit | 1 | 1 | 0.5 | 0.3 |
| Metabolic and nutritional disorders | |  |  |  |
| Edema | 5 | 4 | 2.6 | 1.4 |
| Respiratory system |  |  |  |  |
| Cough | 24 | 12 | 12.4 | 6.6 |
| Dyspnea | 10 | 4 | 5.2 | 2.8 |
| Larygismus | 2 | 0 | 1.0 | 0.6 |
| Pharyngitis | 16 | 12 | 8.3 | 4.4 |
| Rhinitis | 16 | 8 | 8.3 | 4.4 |
| Skin and appendages |  |  |  |  |
| Rashes | 28 | 13 | 14.5 | 7.7 |
| Skin discolor | 1 | 1 | 0.5 | 0.3 |
| Urticaria | 1 | 0 | 0.5 | 0.3 |
| Genitourinary system |  |  |  |  |
| Urine abnormality | 1 | 1 | 0.5 | 0.3 |
| Urine frequency | 1 | 1 | 0.5 | 0.3 |
| Musculoskeletal system |  |  |  |  |
| Muscle cramps | 20 | 11 | 10.3 | 5.5 |
| Special senses |  |  |  |  |
| Eye discomfort | 3 | 3 | 1.5 | 0.8 |
| Tinnitus | 1 | 0 | 0.5 | 0.3 |

Notes:

^a^ Assessment of the adverse events is undertaken using the 1995 ‘Coding Symbols for the Thesaurus of Adverse Reaction Terms’ (COSTART).

^b^ The number of cases was used as the numerator for the calculation of the risk. The denominators for the calculation of the risks were 1,936 person-days and 3,633 person-sachets.
